# Supplementary material for: Single cell RNA-seq and ATAC-seq analysis of cardiac progenitor cell transition states and lineage settlement
Source: Nat Commun. 2018 Nov 19;9:4877. doi: 10.1038/s41467-018-07307-6 (PMC6242939; doi:10.1038/s41467-018-07307-6)
Supplement: Supplementary file 1 — Supplementary Information [file 41467_2018_7307_MOESM1_ESM.pdf]

Supplementary Information

Single cell RNA-seq and ATAC-seq analysis of cardiac progenitor cell transition states and lineage settlement

Jia et al.

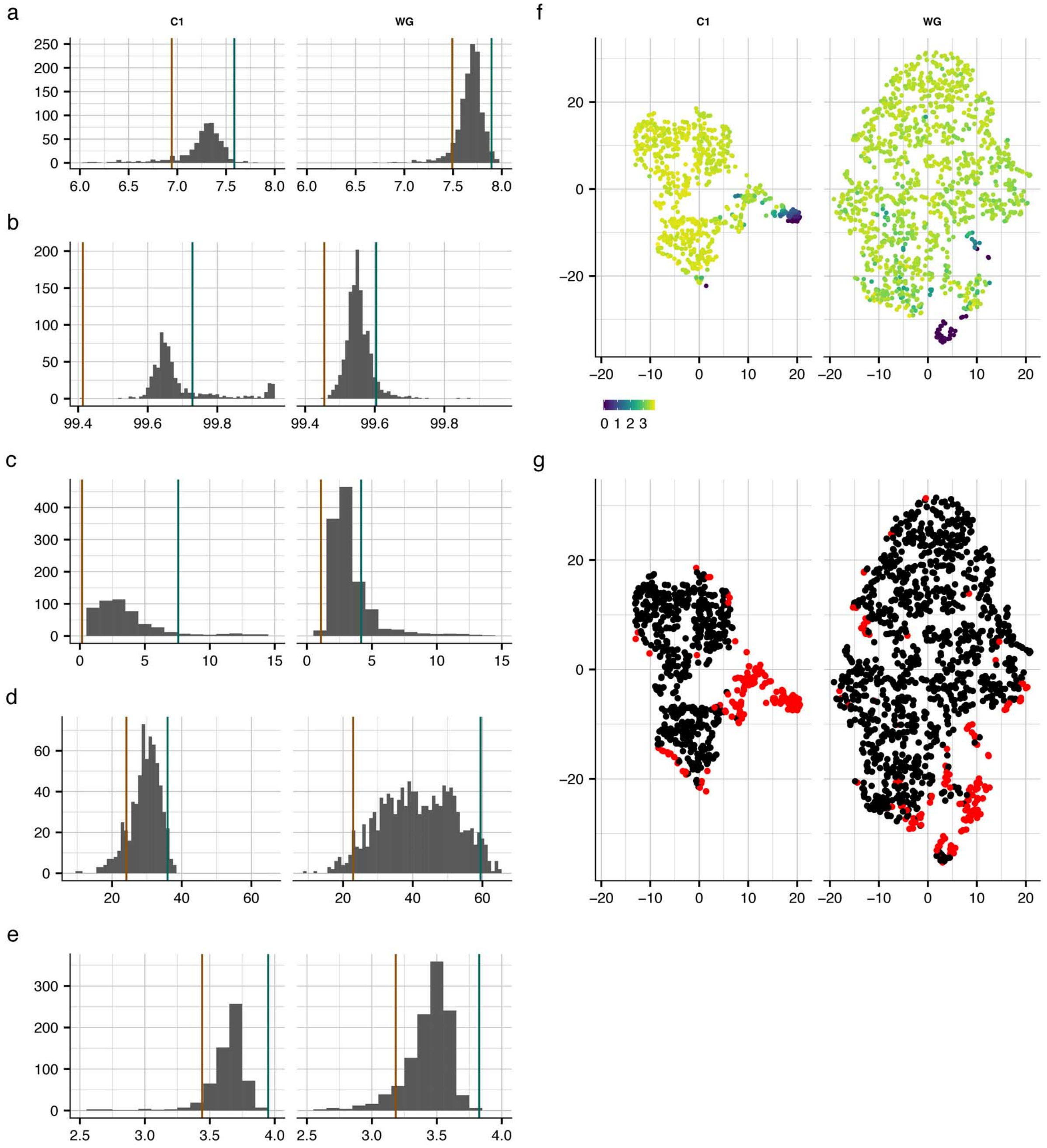

Supplementary Figure 1. Depiction of criteria for cell quality assessment and filtering. (a-e) Distributions of relevant quality criteria for single cells analyzed by the C1 platform (left panel) and Wafergen platform (right panel). The number of detected features (genes, log2 scale) per cell, gene dropout rates per cell, percentage of counts to mitochondrial features, percentage of reads aligned to genes, Rplp0 counts (expression, $\log_{10}$ scale) are shown (in this order). Source data for (a-e) are provided in the Source Data file. The t-SNE projection, based on quality criteria, aggregates cells with similar numbers of detected features in close proximity **(f)** and was used to identify low quality cells (marked in red) in **(g)**.

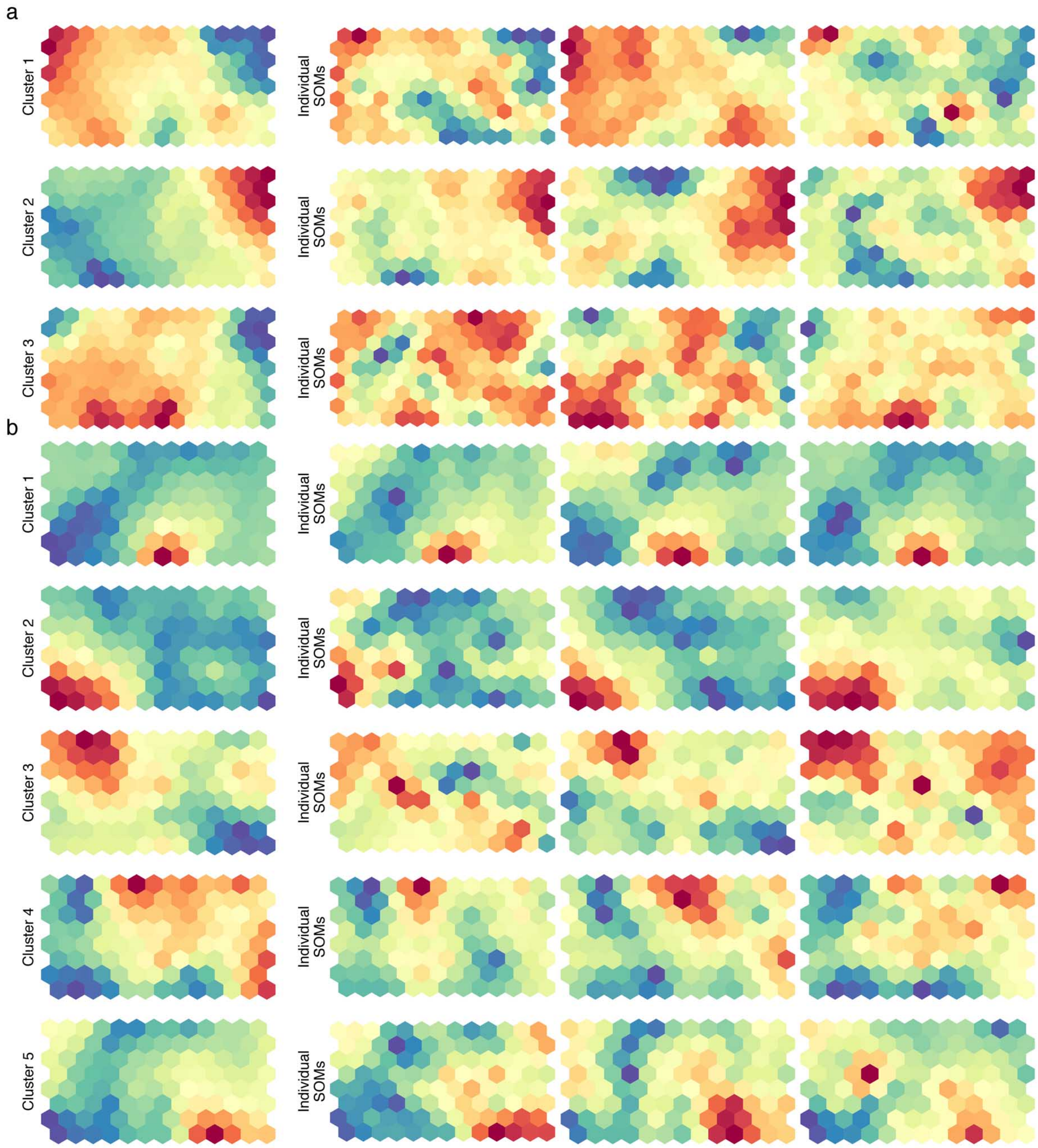

Supplementary Figure 2. Single cell self-organizing maps of scRNA-seq data and clustering. (**a**) Examples of SOM mapping and clustering for Nkx2-5^{+} data. For each cell, an individual SOM (right panel) was created, effectively summarizing the cells transcriptome in reduced dimensionality. Reduced individual cell representations were used for clustering and visualization by t-SNE. Aggregated signals from individual SOMs in clusters resemble key characteristics of individual SOMs (left panel). (**b**) Key characteristics of aggregated and individual SOMs for Isl1^{+} data.

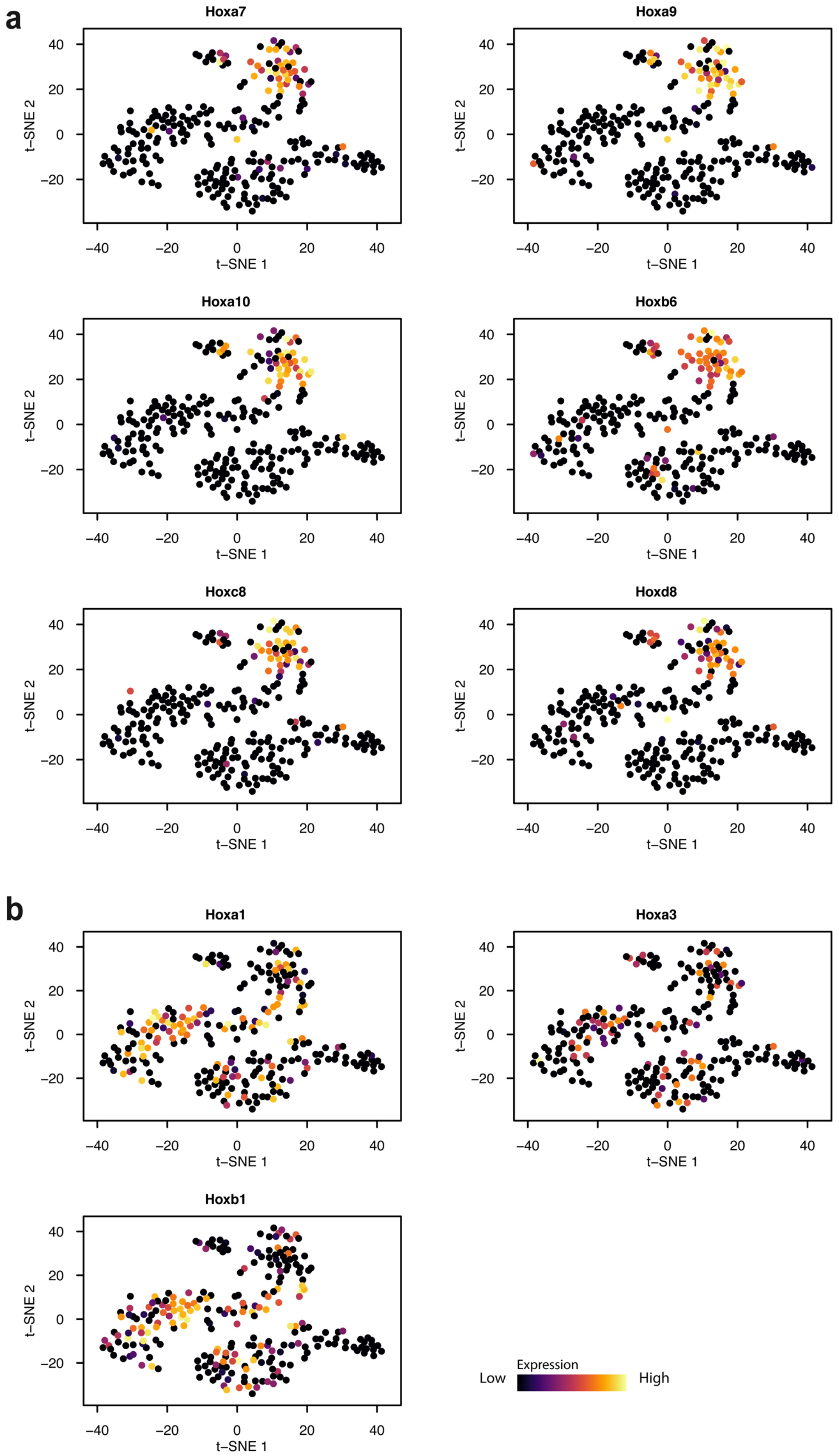
a
Hoxa7
Hoxa9
Hoxa10
Hoxb6
Hoxc8
Hoxd8
b
Hoxa1
Hoxa3
Hoxb1
t−SNE 1
t−SNE 2
Expression
Low
High

Supplementary Figure 3. Expression of posterior Hox genes in a distinct Isl1^{+} CPC subpopulation. t-SNE plots depicting the expression of posterior (**a**) and anterior (**b**) *Hox* genes in distinct clustes of Isl1^{+} CPCs.

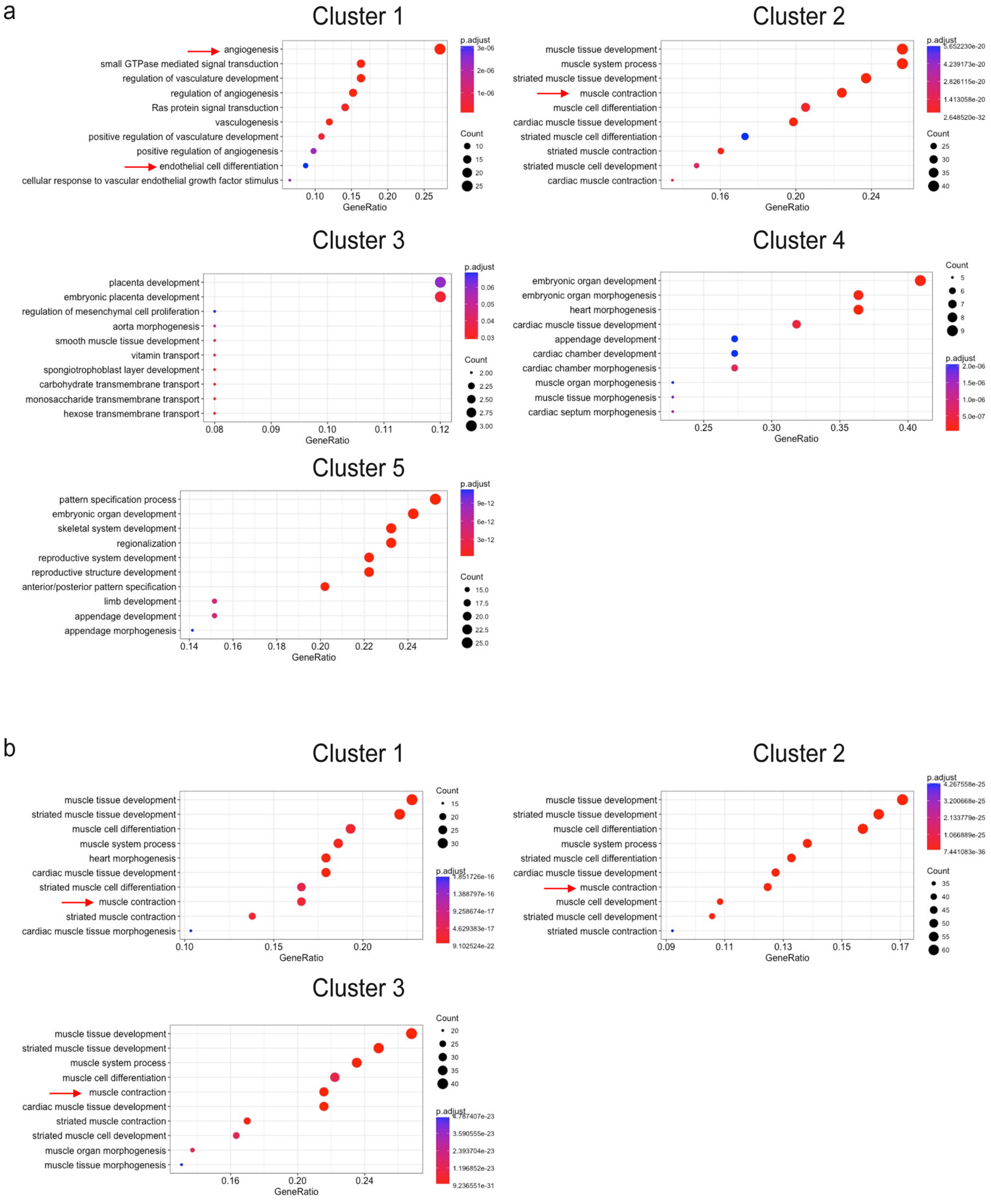

Supplementary Figure 4. Gene Ontology (GO) enrichment analyses of different Isl1⁺ CPC subpopulations. GO enrichment analysis of heterogeneous clusters of Isl1⁺ (**a**) and Nkx2-5⁺ (**b**) CPCs. The relevant GO terms are highlighted by red arrows. Gene enrichment was performed using the R package clusterProfiler.

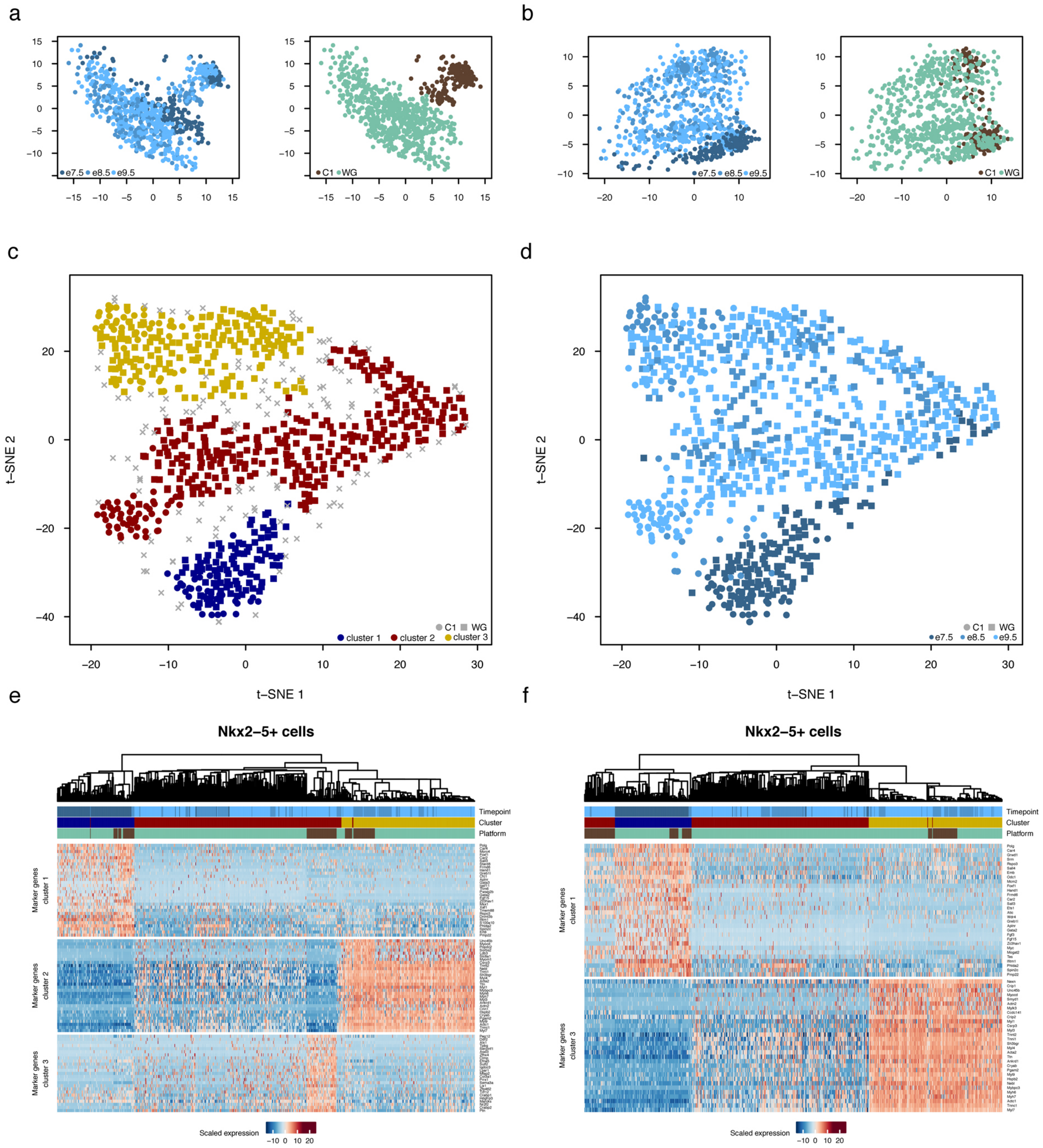

Supplementary Figure 5. Depiction of procedures for correction of batch effects. Single cell alignment and batch correction between two sequencing platforms using data from Nkx2.5$^+$ cells. Principle component analysis (PCA) of **(a)** unaligned single cells indicates batch effects between different sequencing platforms (right), which was diminished by **(b)** alignment of single cells using MnnCorrect. **(c)** t-SNE/HDBSCAN clustering of aligned single cells from the two sequencing platforms identifies three clusters that aggregate by **(d)** capture time point, but not the sequencing platform. Clustering resembles the clustering results when using data from the C1 platform alone

(Figure 1c and 1d, left). **(e)** Heat maps showing expression of the top 30 differentially expressed genes between clusters from Figure 1c using results from both sequencing platforms. Cells in cluster 2 that were processed on the Wafergen system fail to show a consistent expression signature for cluster 2, as compared to cells that were processed on the C1 system. **(f)** Heatmaps showing results from differential expression analysis between clusters from (c). No differentially expressed genes were detected for cluster 2, most likely due to the inconsistent expression signature of cells processed with the Wafergen system. Source data for (a-f) are provided in the Source Data file.

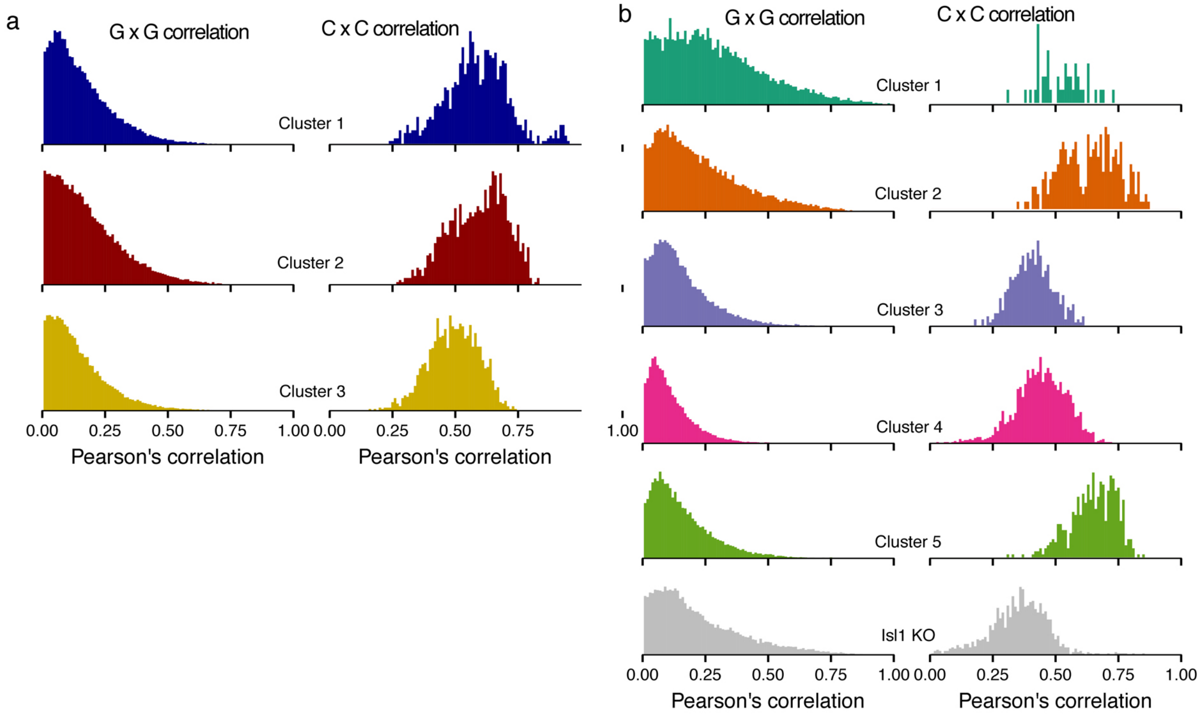

Supplementary Figure 6. Analysis of gene-to-gene and cell-to-cell correlations. Gene-to-gene and cell-to-cell correlations reveal different characteristics of heart development. **(a** and **b)** Pearson coefficients of pairwise gene-gene and cell-cell correlations for clusters from Nkx2-5^{+} (a) and Isl1^{+} (b) cells. Cell-to-cell correlations for Nkx2-5^{+} CPCs are comparable across clusters, while cell-to-cell correlation decreases for Isl1^{+} CPCs in clusters 3, 4 and KO, indicating elevated transcriptional noise in these sub-populations. Source data are provided in the Source Data file.

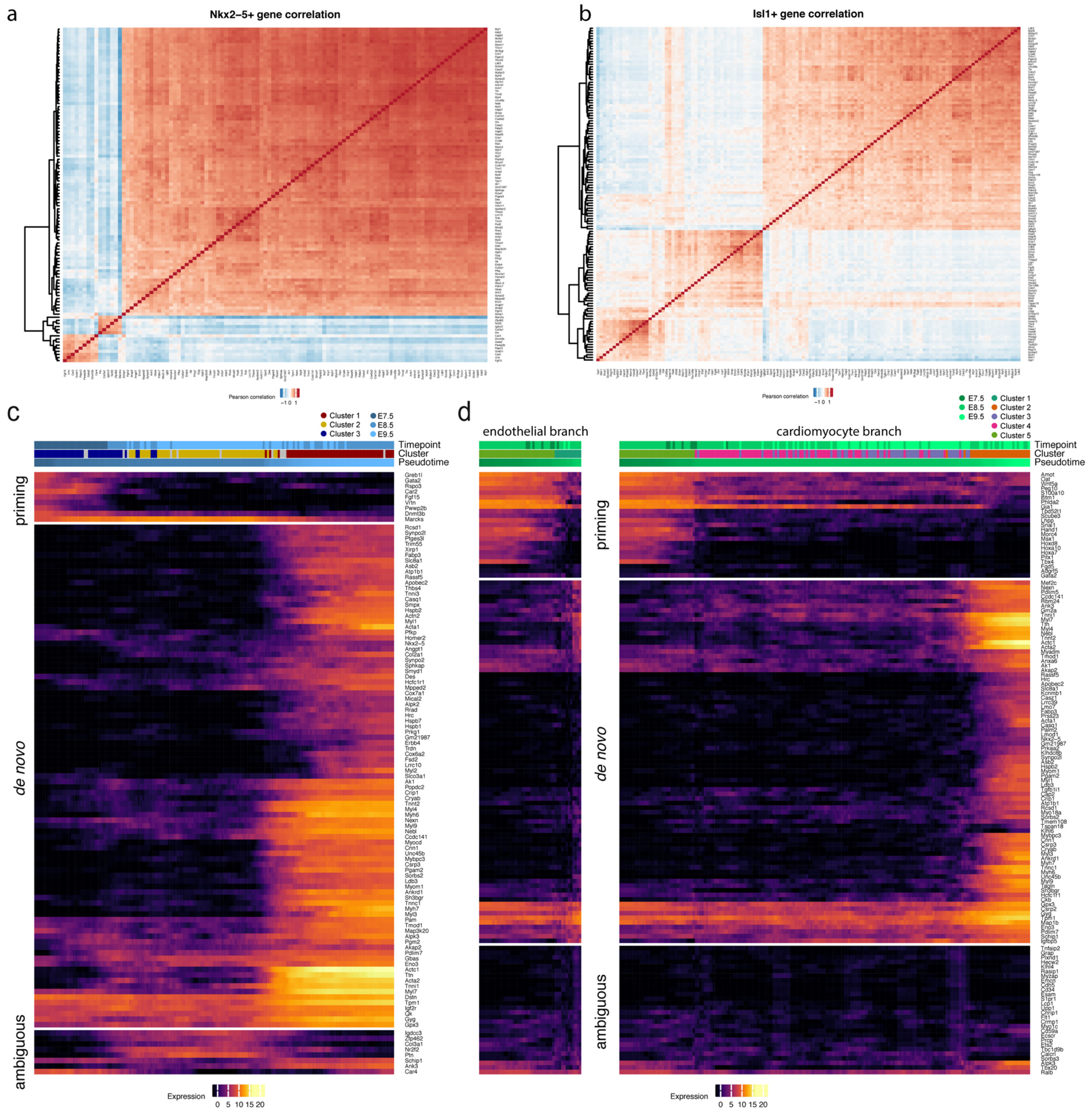

Supplementary Figure 7. Distinct gene sets correlate with developmental pseudotime in Nkx2.5^{+} and Isl1^{+} CPCs. (a, b) Gene-gene correlation matrices for genes affecting the position of Nkx2.5^{+} (**a**) and Isl1^{+} cells (**b**) on developmental trajectories. **(c, d)** Heatmaps showing the expression of genes positively correlated with pseudotime (Spearman rank correlation coefficient > 0.5) in Nkx2-5^{+} cells (**c**) and Isl1^{+} cells (**d**). The two bifurcation trajectories of Isl1^{+} cells are shown separately in (d). Source data for (a-d) are provided in the Source Data file.

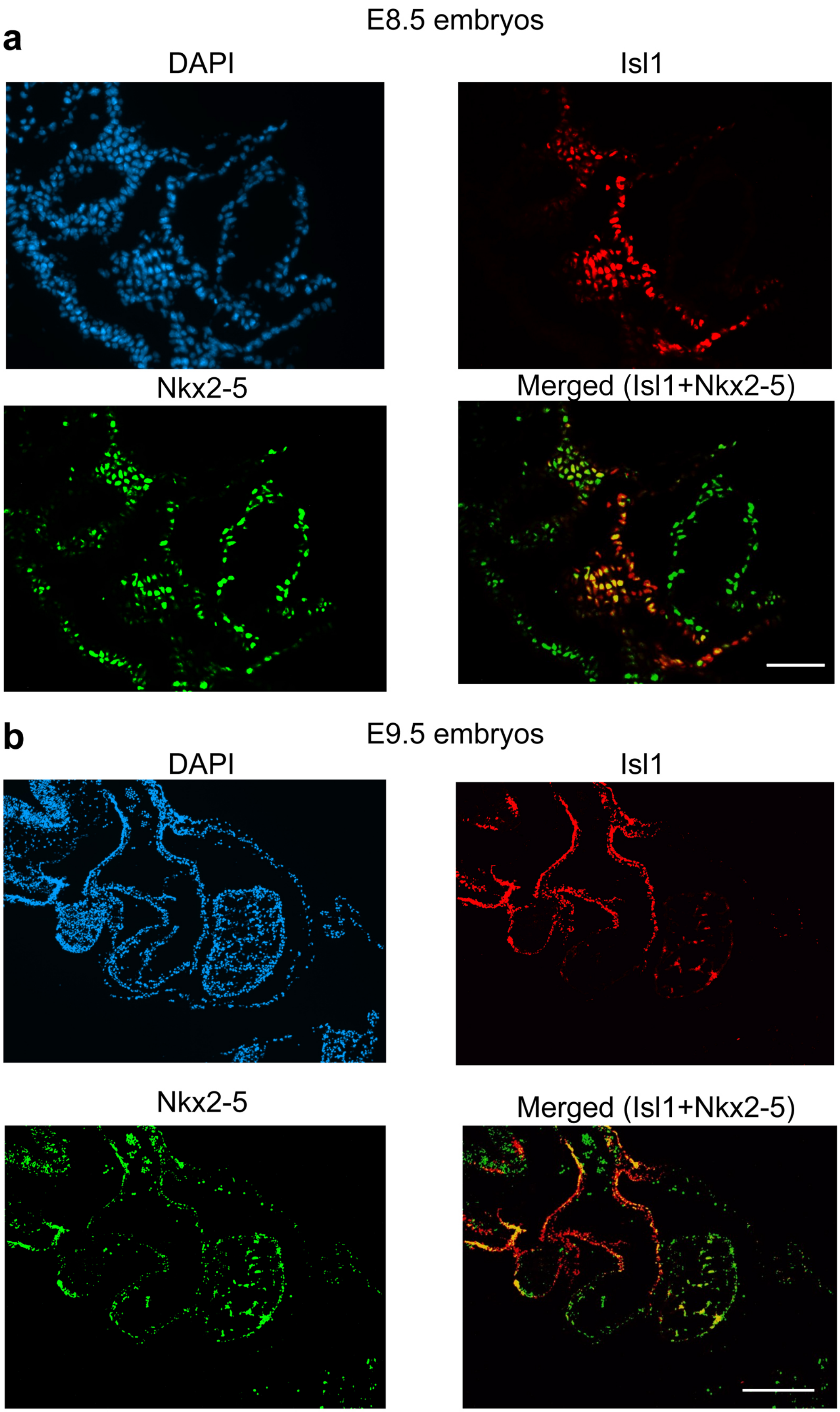
a
E8.5 embryos
DAPI
Isl1
Nkx2-5
Merged (Isl1+Nkx2-5)
b
E9.5 embryos
DAPI
Isl1
Nkx2-5
Merged (Isl1+Nkx2-5)

Supplementary Figure 8. Isl1 and Nkx2-5 are co-expressed in a subset of CPCs in the embryonic heart. (**a, b**) Immunofluorescence images of cryosections from E8.5 (**a**) and E9.5 (**b**) wild type C57/Bl6 embryos stained with Isl1 (red) and Nkx2-5 (green) antibodies. Scale bars: 50 μm for (**a**) and 100 μm for (**b**).

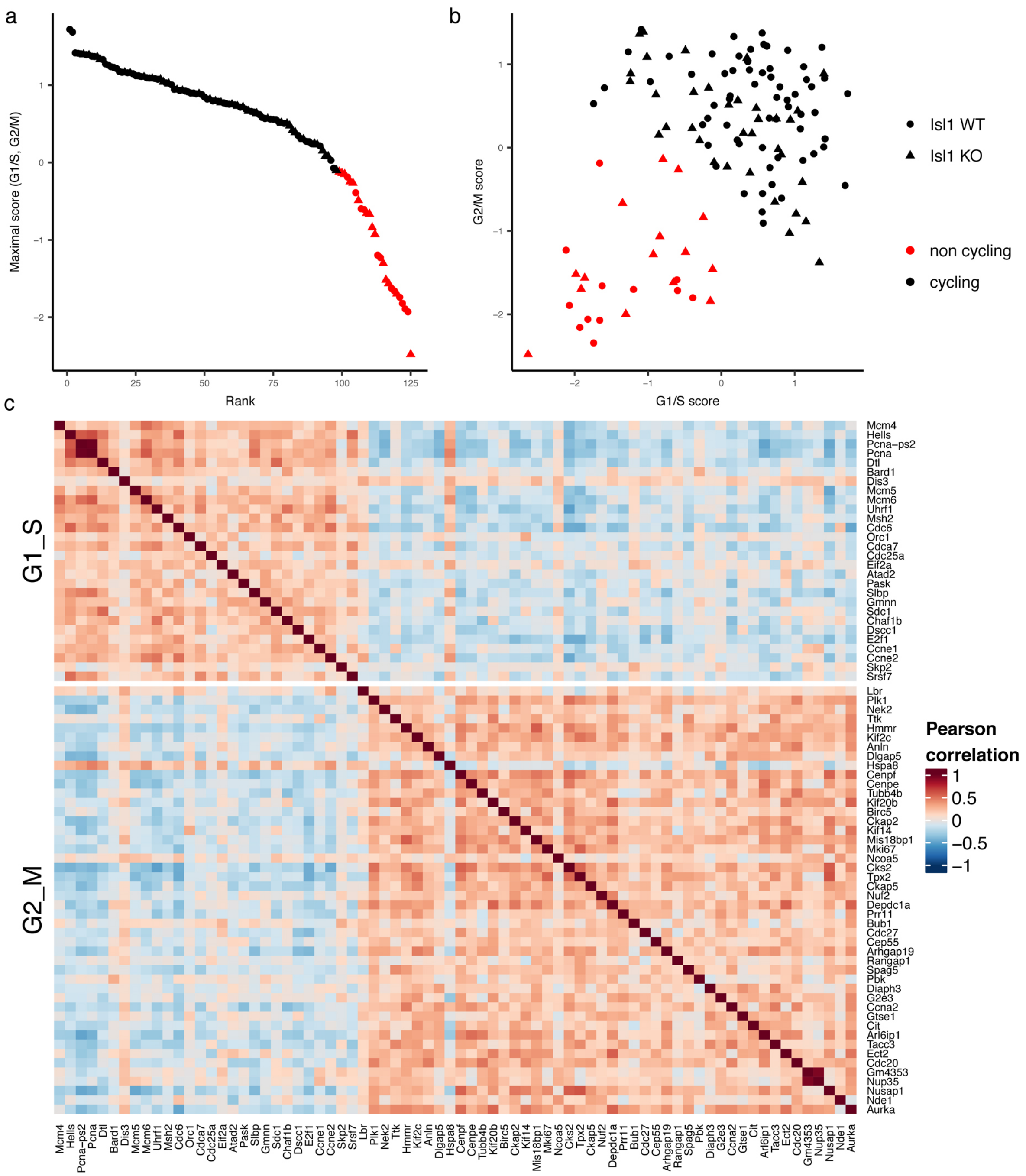

Supplementary Figure 9. Inactivation of Isl1 reduces expression of cell cycle genes in Isl1-GFP$^+$ cells. (**a**) Cells ordered by the maximum of G1/S and G2/M cell cycle scores. Cells with a maximum score lower than -0.1 where considered non-cycling and are marked in red. The cutoff was determined empirically. (**b**) Scatterplot showing G1/S cell cycle score vs. G2/M cell cycle score. Cells identified as cycling in (**a**) accumulate in the first quadrant, indicating their cell cycle status. (**c**) Gene-to-gene correlation of G1/S and G2/M phase genes reveals distinct cell cycle signatures for cycling cells identified in (**a**). Source data are provided in the Source Data file.

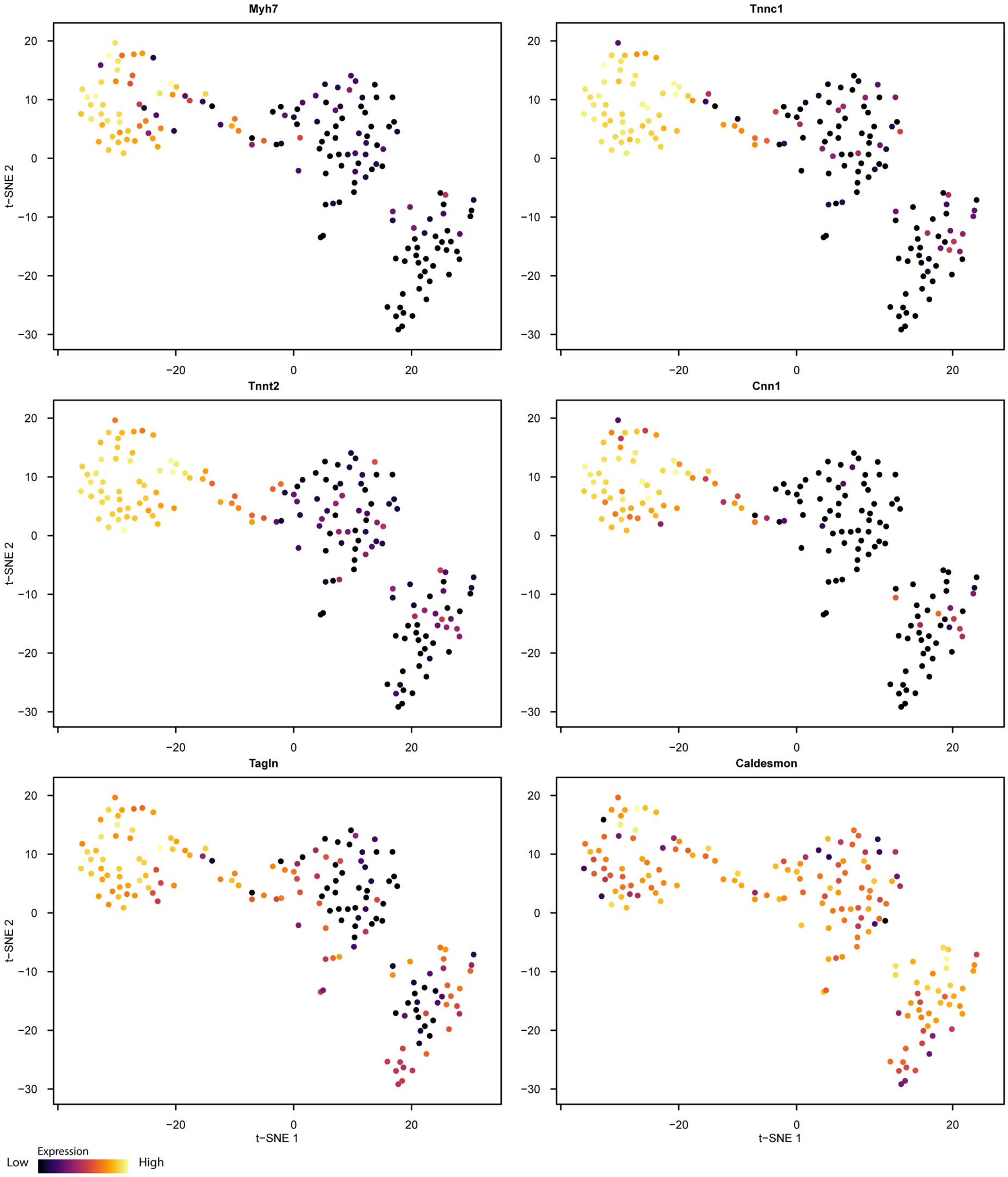

Supplementary Figure 10. Single Nkx2-5⁺ CPCs co-express smooth muscle cell and cardiac markers at E8.5. t-SNE plots showing expression levels of Myh7, Tnnc1, Tnnt2, Cnn1, Tagln, and caldesmon in individual Nkx2-5⁺ CPCs at E8.5 indicating that some Nkx2-5⁺ CPCs co-express smooth muscle cell and cardiac markers.

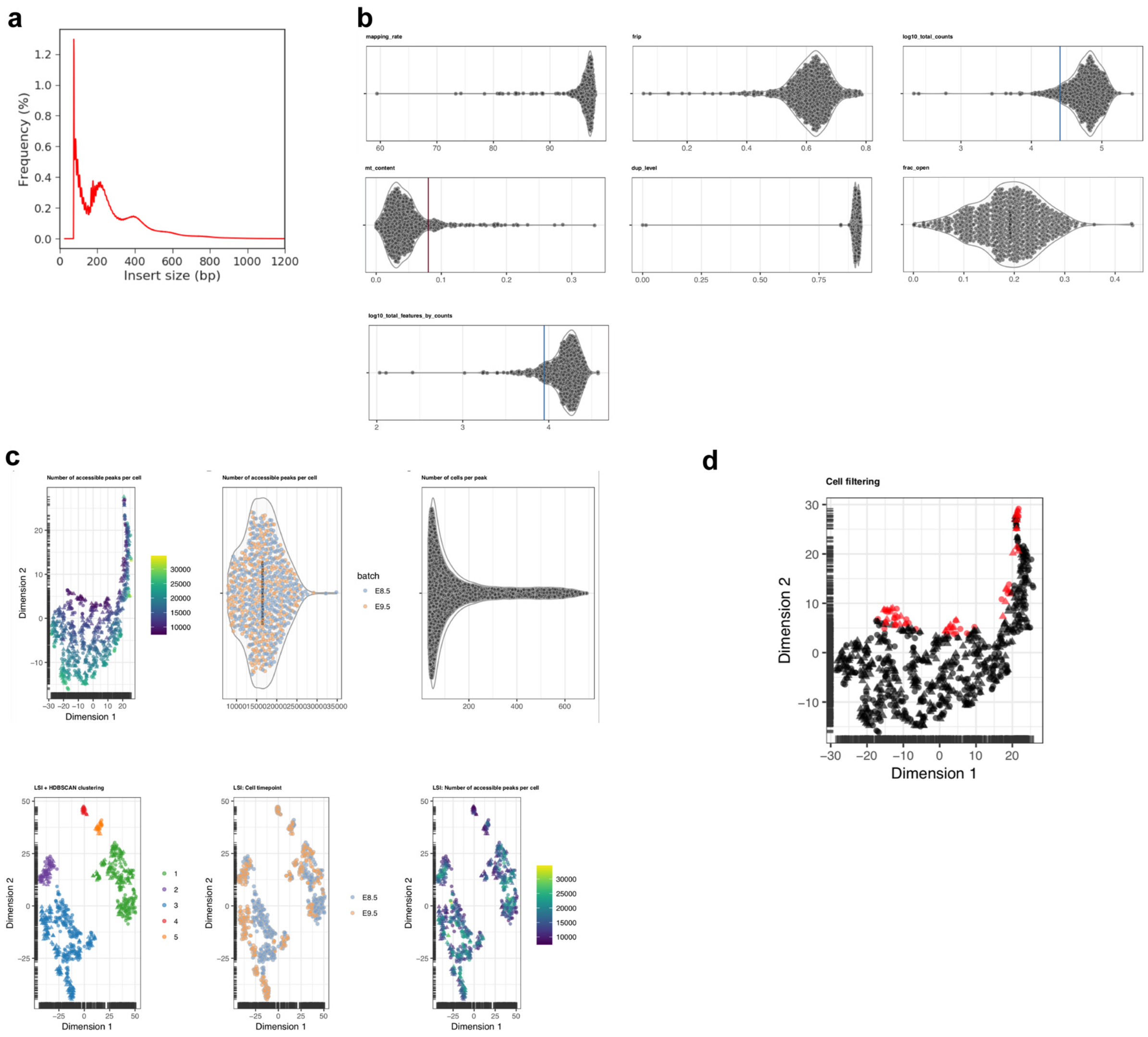

Supplementary Figure 11. Depiction of criteria for quality assessment of single cell ATAC-seq data. (**a**) Distributions of insert size frequencies of libraries from aggregated scRNA-seq. (**b**) Distributions of relevant quality criteria for single cells. Shown are (in order) the mapping rates, fraction of reads in peaks (frip), log10 total counts, mitochondria DNA content, duplication level, fraction of open peaks, number of detected features (log10 scale) per cell. (**c**) The t-SNE projection, based on quality criteria, aggregates cells with similar numbers of detected features and was used to identify low quality cells (marked in red) in **(d)**.

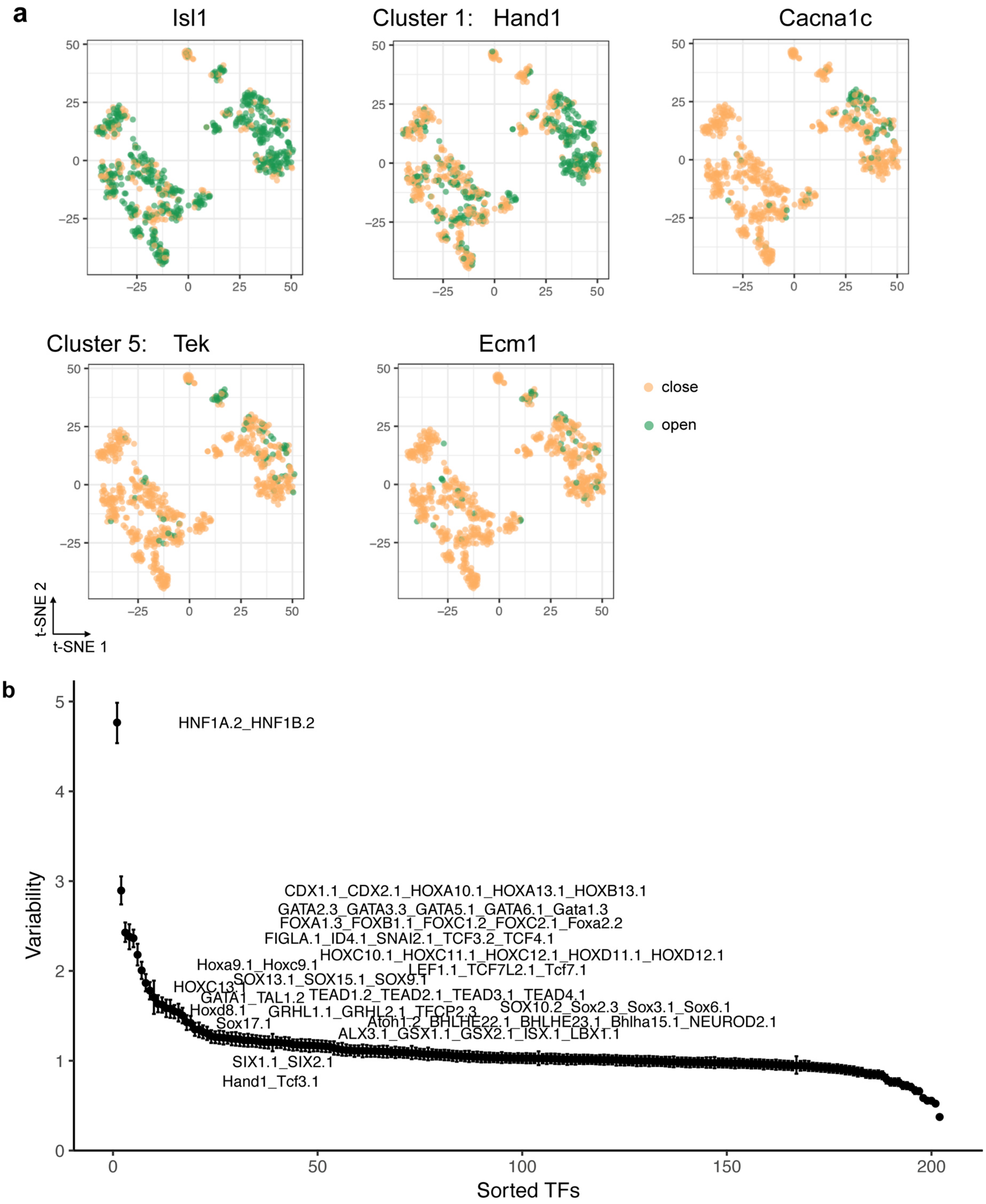

Supplementary Figure 12. Identification of subpopulation-specific open chromatin regions and TF motifs. (**a**) The same t-SNE plot as in Fig. 7b is shown but colored by the number of counts in peaks near indicated marker genes. (**b**) *Z*-scores of transcription motif variability in all single cell chromatin accessibility profiles used in this study.

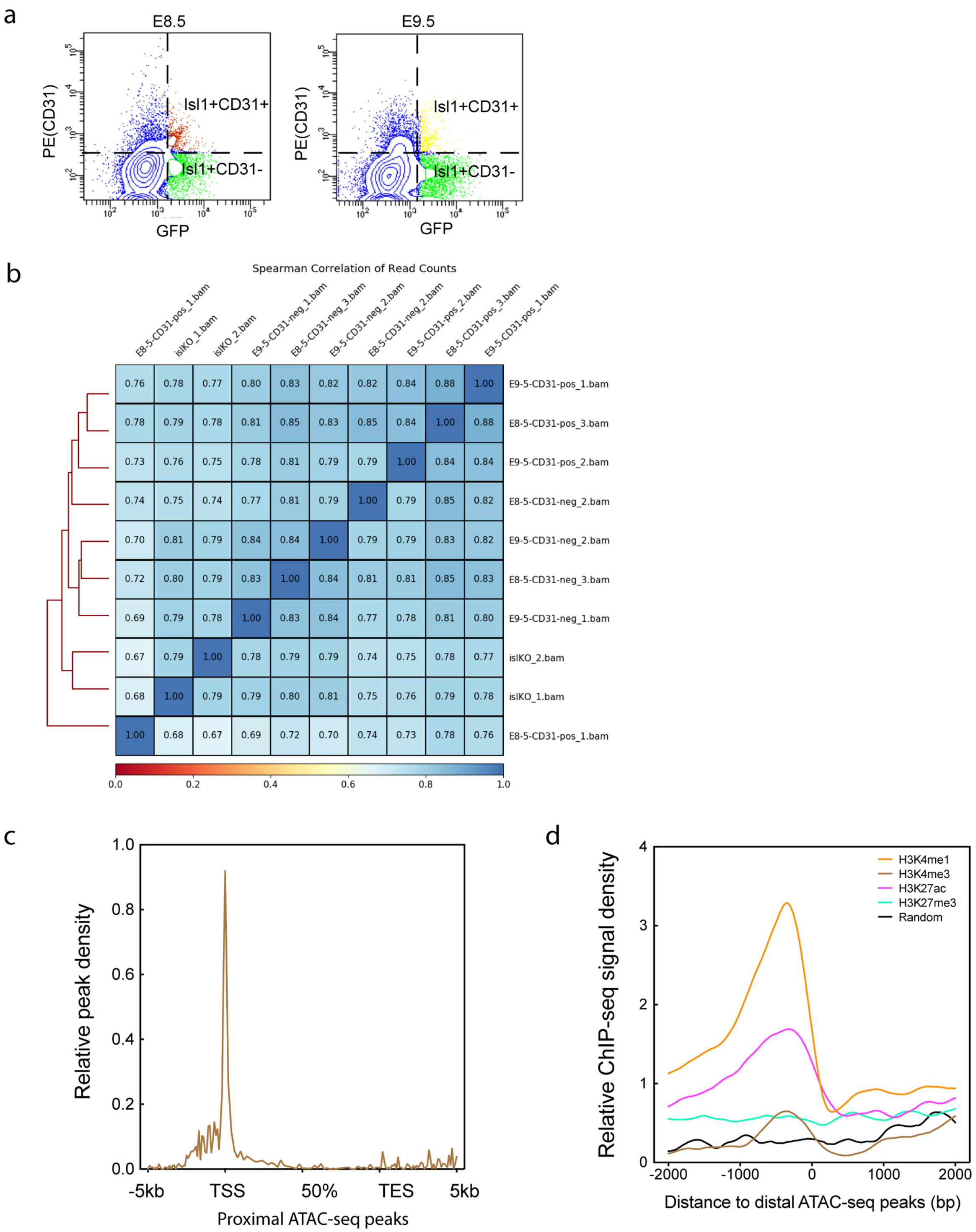

Supplementary Figure 13. Analysis of chromatin configuration in Isl1$^+$CD31$^-$, Isl1$^+$CD31$^+$ and Isl1 KO CPCs by bulk ATAC-seq. (**a**) Sorting strategy for Isl1$^+$CD31$^-$ (cardiomyocyte branch) and Isl1$^+$CD31$^+$ (endothelial branch) for bulk ATAC-seq. (**b**) Heatmap showing Spearman correlation coefficients of biological replicate samples used

in this study. At least two biological replicates with Spearman correlation coefficients above 0.7 were retained for further analysis. (**c**) Metagene plot showing the distribution of distal peaks. The relative enrichment of ATAC-seq signals was aggregated based on relative positions to all genes. (**d**) Distribution of ChIP-seq signal for different histone modifications in E10.5 embryonic hearts across distal ATAC-seq peaks.

a

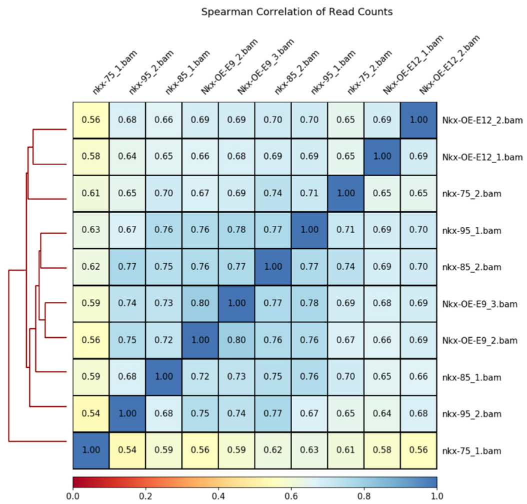

b

Gene ontology analysis of accessible genes in Cluster 1 (n=68)

	Reference	Query (proximal genes)					
GO cellular process	#		# expected	Fold Enrichment	+/-	raw P value	FDR
I band	134	5	.43	11.70	+	8.13E-05	3.16E-02
sarcomere	181	6	.58	10.39	+	2.90E-05	5.64E-02
myofibril	204	6	.65	9.22	+	5.54E-05	3.59E-02
contractile fiber	219	6	.70	8.59	+	8.11E-05	3.94E-02
contractile fiber part	195	6	.62	9.65	+	4.34E-05	4.22E-02

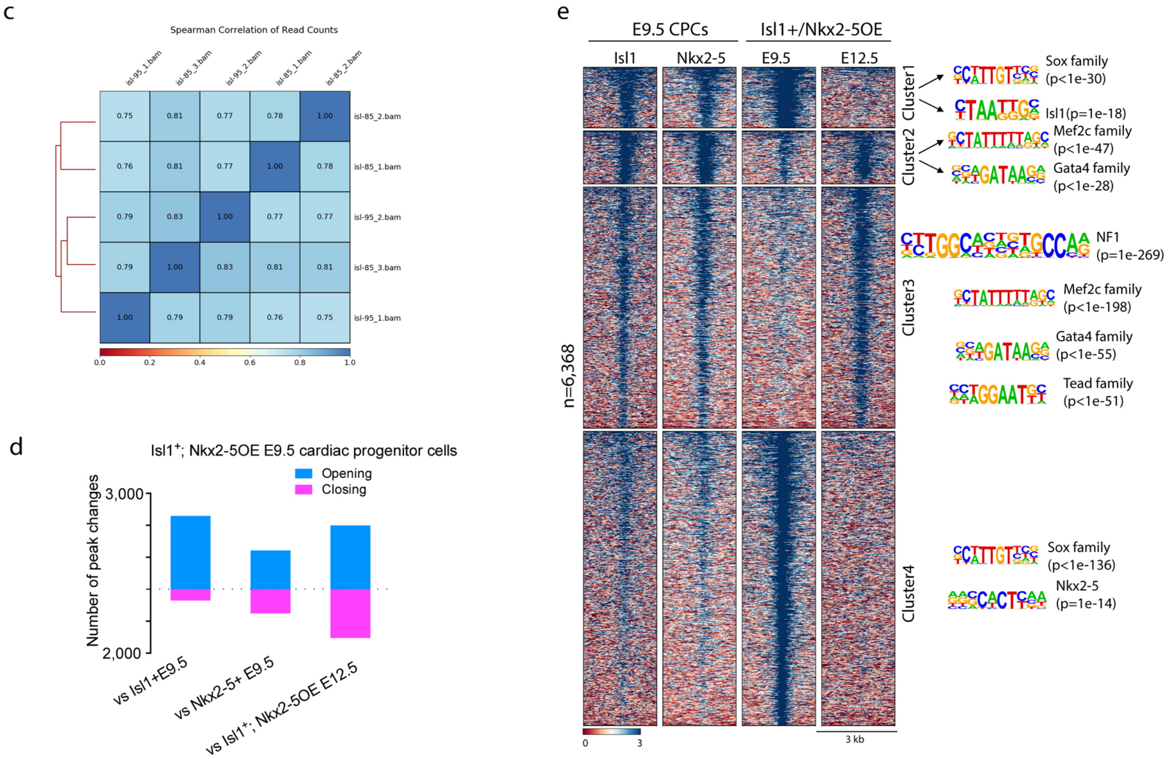

Supplementary Figure 14. Analysis of chromatin configuration in Nkx2-5^{+} CPCs by bulk ATAC-seq. (**a**) Heatmap showing Spearman correlation coefficients of biological replicates used in the study. At least two biological replicates with Spearman correlation

coefficients above 0.6 were employed for further analysis. (**b**) GO enrichment of cluster 1 of Nkx2-5$^+$ CPCs. (**c**) Heatmap showing Spearman correlation coefficients of biological replicate samples of bulk Isl1$^+$ CPCs at E8.5 and E9.5 used for Fig. 7a. (**d**) Number of differential chromatin accessibility peaks (log2(FC)>2, false discovery rate [FDR]<0.05) of Nkx2-5$^+$ overexpression CPCs at E9.5 comparing with Nkx2-5$^+$ and Isl1$^+$ CPCs at E9.5, and Nkx2-5$^+$ overexpression CPCs at E12.5. (**e**) Genome-wide distribution of differential open chromatin peaks in (**d**) grouped by *K*-means. Each row represents one differential peak in sequential comparisons (log2[FC]>2, FDR<0.05). Enriched TF motifs for each cluster are indicated on the right.
