# Supplementary material for: Single cell RNA-seq and ATAC-seq analysis of cardiac progenitor cell transition states and lineage settlement
Source: Nat Commun. 2018 Nov 19;9:4877. doi: 10.1038/s41467-018-07307-6 (PMC6242939; doi:10.1038/s41467-018-07307-6)
Supplement: Supplementary file 4 — Description of Additional Supplementary Files [file 41467_2018_7307_MOESM4_ESM.pdf]

Description of Additional Supplementary Files

Supplementary Data 1. List of genes that are differentially expressed in Nkx2-5$^+$ CPC clusters. Listed are all genes that were found to be up- or downregulated by a log fold change of >2 in cells of a cluster vs. all other cells by MAST (see Methods). Columns are: geneID: the Ensembl Gene ID, pval: the p-value of the estimate, lfc: log fold change, lfc.lo: the lower bound (95% CI) of the lfc, lfc.hi: the higher bound (95% CI) of the lfc, fdr.x: multiple testing corrected p-value, basemeanA: mean gene expression across cells in the cluster, basemeanB: mean gene expression across all other cells, auroc: area under the curve of the gene ranking approach, clusts: cluster for which the gene can be assigned as marker gene, marker_pval: p-value of the gene ranking approach, marker_fdr: multiple testing corrected p-value of the gene ranking approach.

Supplementary Data 2. List of genes that are differentially expressed in Isl1$^+$ CPC clusters. All genes are listed that are up- or downregulated by a log fold change of >2 in cells of a cluster vs. all other cells using MAST (see Methods). Columns are: geneID: the Ensembl Gene ID, pval: the p-value of the estimate, lfc: log fold change, lfc.lo: the lower bound (95% CI) of the lfc, lfc.hi: the higher bound (95% CI) of the lfc, fdr.x: multiple testing corrected p-value, basemeanA: mean gene expression across cells in the cluster, basemeanB: mean gene expression across all other cells, auroc: area under the curve of the gene ranking approach, clusts: cluster for which the gene can be assigned as marker gene, marker_pval: p-value of the gene ranking approach, marker_fdr: multiple testing corrected p-value of the gene ranking approach.

Supplementary Data 3. List of gene correlation analysis results for Nkx2-5$^+$ and Isl1$^+$ cells. Listed are Spearman Rank correlation values for all genes that were differentially expressed (all protein coding genes with an AUROC > 0.8, FDR < 0.01 and a lfc.low > 2 or lfc.hi < -2, FDR < 0.01 from Supplementary Tables 1 and 2). Cluster specific columns contain the local correlation of the gene within the cluster, the dpt column contains the (global) correlation of the genes expression with diffusion pseudotime.

Supplementary Data 4. List of specific peaks obtained by scATAC-seq in each cluster. Specific peaks for each cluster, chromatin coordinators, distance to the nearest genes and the gene symbols, and the q-value of each peak for each cluster are listed.

Supplementary Data 5. List of parameters for quality statistics of processed bulk ATAC-seq samples. Statistic parameter (i.e. number of raw/trimmed reads, mapping statistics from STAR, and results from a read deduplication step to remove PCR amplification artifacts) are listed.

Supplementary Data 6. List of differential chromatin accessibility peaks of Isl1 KO cells. The chromatin coordinator and nearest gene ID of each differential ATAC-seq peak are listed. Differential peaks were unified peaks of E8.5 and E9.5 Isl1 KO compared to Isl1$^+$CD31$^+$ cells.

Supplementary Data 7. List of differential chromatin accessibility peaks of Nkx2-5 CPCs. The chromatin coordinator and nearest gene ID of each differential ATAC-seq peak are listed. The first tab contains all unified differential peaks of E7.5, E8.5 and E9.5 Nkx2-5 CPCs. The second tab contains all unified differential peaks of E9.5 and E12.5 Nkx2-5OE cells compared to E9.5 Isl1^{+} and Nxk2-5^{+} CPCs.
