# Supplementary material for: Single cell RNA-seq and ATAC-seq analysis of cardiac progenitor cell transition states and lineage settlement
Source: Nat Commun. 2018 Nov 19;9:4877. doi: 10.1038/s41467-018-07307-6 (PMC6242939; doi:10.1038/s41467-018-07307-6)
Supplement: Supplementary file 12 — Reporting Summary [file 41467_2018_7307_MOESM12_ESM.pdf]

nature research

Corresponding author(s): Thomas Braun

Reporting Summary

Nature Research wishes to improve the reproducibility of the work that we publish. This form provides structure for consistency and transparency in reporting. For further information on Nature Research policies, see Authors & Referees and the Editorial Policy Checklist.

Statistical parameters

When statistical analyses are reported, confirm that the following items are present in the relevant location (e.g. figure legend, table legend, main text, or Methods section).

n/a	Confirmed	
☐	☒	The exact sample size (*n*) for each experimental group/condition, given as a discrete number and unit of measurement
☐	☒	An indication of whether measurements were taken from distinct samples or whether the same sample was measured repeatedly
☐	☒	The statistical test(s) used AND whether they are one- or two-sided<br>*Only common tests should be described solely by name; describe more complex techniques in the Methods section.*
☒	☐	A description of all covariates tested
☐	☒	A description of any assumptions or corrections, such as tests of normality and adjustment for multiple comparisons
☐	☒	A full description of the statistics including central tendency (e.g. means) or other basic estimates (e.g. regression coefficient) AND variation (e.g. standard deviation) or associated estimates of uncertainty (e.g. confidence intervals)
☐	☒	For null hypothesis testing, the test statistic (e.g. F, t, r) with confidence intervals, effect sizes, degrees of freedom and P value noted<br>*Give P values as exact values whenever suitable.*
☒	☐	For Bayesian analysis, information on the choice of priors and Markov chain Monte Carlo settings
☒	☐	For hierarchical and complex designs, identification of the appropriate level for tests and full reporting of outcomes
☐	☒	Estimates of effect sizes (e.g. Cohen's d, Pearson's r), indicating how they were calculated
☐	☒	Clearly defined error bars<br>*State explicitly what error bars represent (e.g. SD, SE, CI)*

Our web collection on statistics for biologists may be useful.

Software and code

Policy information about availability of computer code

Data collection	Images: The software ZEN pro associated with Zeiss Z1 and LSM800 microscope was used to obtain images.<br>Sequencing: The NextSeq System Suite software associated with NextSeq 500 was used to collect raw sequencing data.<br>FACS: FACSDiva software associated with BD Arial II was used to collect FACS data.
Data analysis	We used the following software: R3.5.4 and R packages: scater1.8.3, MAST1.6.1, SC3_1.8.0, SingleCellExperiment1.2.0, chromVAR1.2.0, DESeq2_1.20.0, edgeR3.22.3. Other software: trimmomatic-0.33, STAR 2.6, Samtools1.9, deepTools2, MACS2, Picard2.18.11, bedTools2.27.0, Homer_v4.10. The latest available software version was used for the analysis. The custom code (R scripts) used for data analysis and simulations are freely available on request from the corresponding author or be downloaded from [https://github.com/loosolab/cardiac-progenitors].

For manuscripts utilizing custom algorithms or software that are central to the research but not yet described in published literature, software must be made available to editors/reviewers upon request. We strongly encourage code deposition in a community repository (e.g. GitHub). See the Nature Research guidelines for submitting code & software for further information.

Data

Policy information about availability of data

All manuscripts must include a data availability statement. This statement should provide the following information, where applicable:

- Accession codes, unique identifiers, or web links for publicly available datasets
- A list of figures that have associated raw data
- A description of any restrictions on data availability

All raw and processed data is freely available from the ENA repository [https://www.ebi.ac.uk/ena] under accession number PRJEB23303.

Field-specific reporting

Please select the best fit for your research. If you are not sure, read the appropriate sections before making your selection.

☒ Life sciences ☐ Behavioural & social sciences ☐ Ecological, evolutionary & environmental sciences

For a reference copy of the document with all sections, see nature.com/authors/policies/ReportingSummary-flat.pdf

Life sciences study design

All studies must disclose on these points even when the disclosure is negative.

Sample size	No statistical method was used to predetermine sample size. For scRNA-seq on the Fluidigm C1 system, 291 Isl1+ CPCs and 221 Nkx2-5+ CPCs were collected. We additionally analyzed 767 Nkx2-5+ CPCs using the Wafergen iCell8system. For scATAC-seq, we analyzed Isl1+ CPC at two different time points (E8.5 and E9.5) using 695 cells that had passed quality filtering. CPCs were isolated by FACS. For ATAC-seq and RNA-seq analysis, two replicates were used for each sample. CPCs were isolated from a total number of 403 staged mouse embryos.
Data exclusions	The scRNA-seq and scATAC-seq samples of low quality were excluded as explained in the "Methods" section and supplementary Fig1 & 11. Essentially, we excluded cells that had few reads or few associated features. For bulk ATAC-seq, we used the Spearman method to analyze correlation and reproducibility of replicate samples. We excluded samples, in which the Spearman correlation was lower than 0.6. After exclusion, we used at least 2 replicates for each condition (supplementary Fig13b & 14a), which fulfilled the statistic criteria for downstream analysis.
Replication	For scRNA-seq and scATAC-seq, data from hundreds of cells were collected for each development timepoint, providing replicate measurements for all different conditions/cell sub-populations. For bulk ATAC-seq and RNA-seq, two biological replicates were used for each sample.
Randomization	In cases when embryos with different genotypes were compared, random assignment is not possible (a mutant animal cannot be “randomly” assigned to a wildtype group). One major goal of the study was to analyze how inactivation or overexpression of a gene affects the transcriptome of individual cardiac progenitor cells. CPCs were isolated by activity (fluorescence) of reporter genes for specific experiments, which also prevents randomization. However, cells at each time point were processed in parallel and thus were highly randomized.
Blinding	The investigators were not blinded in regard to allocation of samples during experiments and outcome assessment. For scRNA-seq and scATAC-seq analysis, unsupervised machine learning was applied, and no prior knowledge was used to determine different clusters.

Reporting for specific materials, systems and methods

Materials & experimental systems

n/a	Involved in the study
☒	☐ Unique biological materials
☐	☒ Antibodies
☒	☐ Eukaryotic cell lines
☒	☐ Palaeontology
☐	☒ Animals and other organisms
☒	☐ Human research participants

Methods

n/a	Involved in the study
☒	☐ ChIP-seq
☐	☒ Flow cytometry
☒	☐ MRI-based neuroimaging

Antibodies

Antibodies used	CD31 PE conjugated antibody, for FACS-sorting, 1 ug/ml (BD Pharmingen cat# 553373, clone: 390, lot# E07125-1633)

anti-GFP antibody, for immunofluorescent staining, 1: 2,000 (ThermoFisher Scientific cat# A11120, clone: 3E6, lot# 40351A)
anti-Nkx2-5, for immunofluorescent staining, 1:1,000 (ThermoScientific cat# PA5-49431, lot# TB2526191C)
anti-Isl1, for immunofluorescent staining, 1:100 (DSHB 39.4D5, lot# 8/4/16)

Validation

The CD31 PE conjugated antibody is widely used and was validated previously (e.g., PMID: 18546599). The anti-GFP antibody was used in more than 352 published papers according to information provided by the manufacturer. The anti-Nkx2-5 and anti-Isl1 antibodies were initially described and validated in PMID: 28256502 and 28436940, respectively.

Animals and other organisms

Policy information about studies involving animals; ARRIVE guidelines recommended for reporting animal research

Laboratory animals

All mouse strains with different genotypes were kept on a C57BL/6J genetic background. Mice at 8-24 weeks were used for timed matings to obtain staged embryos. Each embryo was staged by counting the number of somites. The sex of embryos was not determined prior to analysis.

Wild animals

Not involved in this study.

Field-collected samples

Not involved in this study.

Flow Cytometry

Plots

Confirm that:

- [x] The axis labels state the marker and fluorochrome used (e.g. CD4-FITC).
- [x] The axis scales are clearly visible. Include numbers along axes only for bottom left plot of group (a 'group' is an analysis of identical markers).
- [x] All plots are contour plots with outliers or pseudocolor plots.
- [x] A numerical value for number of cells or percentage (with statistics) is provided.

Methodology

Sample preparation

For flow cytometry of Isl1-GFP and Nkx2-5-GFP positive cardiac progenitor cells, samples were directly loaded into the BD FACSAria II and sorted using the GFP channel. To harvest Isl1+CD31- and Isl1+CD31+ CPCs, cells were stained for 30 min on ice using anti-mouse CD31 PE conjugated antibody (BD Pharmingen Cat# 553373) with a concentration of 1ug/ml in the presence of 1% sodium azide, washed extensively with 1×PBS, and sorted using the GFP and PE channels of the BD FACSAria II instrument to isolate GFP and PE double positive or GFP-positive and PE negative cells.

Instrument

BD FACSAria II

Software

BD FACSDiva Software. No custom code was used in this study.

Cell population abundance

The respective information is provided in Supplementary Fig. 13a.

Gating strategy

The respective information is provided in Supplementary Fig. 13a.

- [x] Tick this box to confirm that a figure exemplifying the gating strategy is provided in the Supplementary Information.
